# Fitness Trade-Offs and Potential Metabolic Resistance Mechanisms in Geographically Distinct Strains of *Trichogramma dendrolimi*: Implications for Imidacloprid Resistance Management

**DOI:** 10.3390/insects16101038

**Published:** 2025-10-09

**Authors:** Yu-Tong Li, Xiang-Xin Kong, Wu-Nan Che, Jin-Cheng Zhou, Shu-Qi Wang, Hui Dong

**Affiliations:** 1 College of Plant Protection, Shenyang Agricultural University, Shenyang 110866, China; 15998232038@163.com (Y.-T.L.); byndkxx@163.com (X.-X.K.);; 2 College of Agriculture and Forestry, Linyi University, Linyi 276000, China; 3 Key Laboratory of Natural Enemies Insects, Ministry of Agriculture and Rural Affairs, Jinan 250100, China

**Keywords:** parasitoids, *Trichogramma dendrolimi*, biological control, geographic strains, transcriptome analysis

## Abstract

**Simple Summary:**

This study helps understand the phenotypic and molecular basis of imidacloprid resistance in *Trichogramma dendrolimi*. The tolerant FS strain has distinct biological trade-offs including less fecundity but greater survival when exposed to insecticide. According to transcriptomic and RT-qPCR analyses, *CYP4C1*, *CYP6K1*, and *GstS1* were found to play a role in detoxification and can be potential targets for resistance monitoring and management. The findings will help in enhancing the understanding of resistance evolution and strategies for sustainable pest control.

**Abstract:**

The widespread use of neonicotinoid insecticides has led to increasing resistance in non-target organisms, including the egg parasitoid *Trichogramma dendrolimi*, a crucial biological control agent. Film-residue bioassays on 17 geographic strains revealed striking inter-strain variability in susceptibility to imidacloprid, with mortality at a discriminating dose of 0.1 mg/L ranging from 25.7% to 87%. The most tolerant (FS) and least tolerant (HA) strains were subsequently selected for evaluation of biological parameters and comparative transcriptomics. Tolerant strains (FS) showed adaptive trade-offs: extended longevity (5.47 ± 0.57 d) and emergence (93.6 ± 1.9%), but reduced fecundity (54.6 ± 4.9 eggs) compared to HA. Transcriptome analysis revealed 2115 differentially expressed genes, with GO enrichment highlighting metabolic and detoxification pathways. KEGG analysis the most enriched pathways were “Protein digestion and absorption” and “Neuroactive ligand-receptor interaction”. RT-qPCR confirmed overexpression of *CYP4C1*, *CYP6K1*, and *GstS1* in FS, indicating their potential roles in metabolic resistance if present. This study presents preliminary evidence of potential fitness trade-offs and molecular mechanisms that could underly imidacloprid resistance in *T. dendrolimi*, which may lead to important insights for resistance monitoring and more sustainable integrated pest management strategies.

## 1. Introduction

The substantial and often increasing reliance on chemical insecticides in many agricultural systems, particularly in developing regions, has led to widespread environmental and ecological concerns, particularly regarding non-target species [1,2]. Among these, neonicotinoids, a class of systemic insecticides, have been extensively used due to their high efficacy against sucking and chewing pests [3]. Nonetheless, their persistence in the environment and sublethal effects on beneficial insects including pollinators and natural enemies, have raised many ecological and regulatory concerns [4,5]. *Trichogramma* wasps, particularly *T. dendrolimi*, are widely used against lepidopteran pests, particularly in forestry, orchards, and grapevines [6,7]. Given their ecological importance, it is important to know their susceptibility and resistance mechanisms to insecticides for management.

Neonicotinoids, including imidacloprid, are broad-spectrum insecticides which function as modular nAChR agonist to overstimulate the nervous systems of target pests [8], and their systemic and residual properties make them potentially harmful to natural enemies including parasitic wasps [9]. Prior studies concerning resistance assessment in wasps of the genus Trichogramma have largely focused on conventional insecticides like methomyl, deltamethrin, phoxim and pyrethroids [10,11,12,13]. Nevertheless, limited research exists regarding toxicity of imidacloprid to *Trichogramma* spp. As a highly effective neurotoxic insecticide, imidacloprid has become one of the world’s most widely used insecticides due to its broad-spectrum efficacy and long residual activity [14]. It controls target pest in the field but at the same time, it affects non-target beneficial insects [1]. As a result, exploring the imidacloprid resistance mechanisms in *T. dendrolimi* is crucial for assessing its ecological risk and developing strategies to conserve its biological control potential within integrated pest management (IPM) frameworks.

Insects usually develop resistance through increased metabolic detoxification by cytochrome P450 monooxygenases (P450s), glutathione S-transferases (GSTs), and esterases [15]. The existing similarity in the mechanisms of insecticide resistance in natural enemies and targeted pests provides a theoretical foundation and practical guidance for studies on resistance development in *T. dendrolimi* [16]. Mechanisms of resistance can impose fitness costs, which affect various life-history traits including fecundity, longevity and developmental time [17,18]. For example, in *Spodoptera litura*, resistance to pyrethroids is caused due to P450 which reduces reproductive output [19]. According to Parreira et al. [20], the parasitism efficiency that affects the success of biocontrol in Trichogramma may change. Measuring these trade-offs is crucial to evaluate the stability of resistance in field populations and for the optimal release of parasitoids.

The objective of this study is to systematically investigate imidacloprid resistance in the ecologically vital parasitoid *T. dendrolimi*. We find notable variation geographic variation in susceptibility through bioassays for 17 strains. Our transcriptomic analyses reveal 2115 differentially expressed genes, with functional enrichment in metabolic pathways, and subsequent RT-qPCR validation confirms the pivotal roles of *CYP4C1*, *CYP6K1*, and *GstS1* in metabolic resistance.

## 2. Materials and Methods

### 2.1. Insects and Reagent

The 17 *T. dendrolimi* populations were initially established from single male–female pairs collected from distinct geographical locations (Table 1). All geographic strains were maintained in the laboratory for over 20 generations on eggs of the factitious host, *Corcyra cephalonica* (Lepidoptera: Pyralidae), under standard rearing conditions (25 ± 1 °C, 70 ± 5% RH, L16:D8 photoperiod) without any exposure to insecticides. The colony of *C. cephalonica* Stainton was obtained from the Biological Control Laboratory of Shenyang Agricultural University. The insects were reared on an artificial diet consisting of a mixture of cornmeal and wheat bran under controlled laboratory conditions. Technical-grade imidacloprid (97% purity; Sino-Agri United Co., Ltd., Beijing, China) was dissolved in acetone to prepare a stock solution, which was subsequently diluted to desired concentrations for bioassays.

### 2.2. Toxicity Bioassay of Imidacloprid in Trichogramma Dendrolimi

Bioassays were conducted using the insecticide-coated tube method [21]. The HR strain, a well-characterized laboratory reference strain, was selected for the initial dose–response bioassay to establish a baseline LC_50_ for the species. An initial dose–response bioassay was conducted using only the HR strain with four concentrations of imidacloprid (0.01, 0.1, 0.5, and 1.0 mg/L) to determine the concentration range inducing minimum survival and mortality. Based on the LC_50_ value of the HR strain (Table 2), a discriminating dose of 0.1 mg/L was established. This dose was chosen not as a diagnostic dose to kill 99% of susceptible individuals, but as an intermediate dose to efficiently rank the relative susceptibility of the 17 geographic strains. This single dose was then used to screen the susceptibility of the 17 geographic strains of *T. dendrolimi* using the film-residue bioassay method. For each concentration or the discriminating dose, along with blank controls (distilled water and 0.1% Triton X-100 (Sangon Biotech Co., Ltd., Shanghai, China)), the tests were replicated three times. Insecticide solutions were applied to 5 mL centrifuge tubes to create treated surfaces. Based on the LC_50_ value of the HR-Td strain, a discriminating dose was established to differentiate between resistant and susceptible individuals in subsequent bioassays. Newly emerged females (<24 h old) were individually exposed (50 wasps per treatment) in treated tubes for 1 h, then transferred to clean tubes with 10% honey solution and maintained in climate-controlled chambers (25–27 °C).

### 2.3. Biological Assays of Trichogramma Dendrolimi

Based on toxicity screening results, tolerant (FS) and susceptible (HA) strains of *T. dendrolimi* were selected for further biological assessment. Fresh *Corcyra cephalonica* eggs were provided as hosts, and parasitoids were reared to adulthood in climate-controlled chambers (25 ± 1 °C, 70 ± 5% RH, 16L:8D photoperiod). Newly emerged mated females were exposed to insecticide-treated surfaces (via the coated-tube method) for 1 h. Each female was transferred to a glass vial (2.5 × 8 cm) containing an egg card (~100 *C. cephalonica* eggs on a 0.5 × 0.5 cm paper square) for parasitism. Vials were sealed with cotton plugs and returned to the incubator (30 replicates per strain). Egg cards were replaced every 24 h, and honey solution (10% *v*/*v*) was provided. Mortality was recorded daily (adult longevity) and deceased wasps were preserved in 75% ethanol (stored at −20 °C for subsequent morphometric analysis). After complete offspring emergence and parental death, the following parameters were quantified under a stereomicroscope: fecundity rate, emergence rate, developmental time, body size, and female ratio.

### 2.4. RNA Extraction and cDNA Library Construction

Newly emerged female *T. dendrolimi* (≤24 h post-eclosion) from both tolerant (FS) and susceptible (HA) strains were pooled into three biological replicates (60 individuals per replicate) and immediately flash-frozen in liquid nitrogen (−80 °C). Sequencing and subsequent analyses were conducted by a specialized company (Frasergen, Wuhan, China), ensuring high-quality data processing and accurate interpretation of the results. Total RNA was extracted using TRIzol reagent (Invitrogen, Waltham, MA, USA), followed by rigorous quality assessment. Measured via NanoDrop 2000 spectrophotometer (Thermo Fisher Scientific, Waltham, MA, USA) (A260/A280 ratio ≥ 1.8; A260/A230 ≥ 2.0). Evaluated using RNA Nano 6000 Assay Kit on an Agilent Bioanalyzer 2100 system (Agilent Technologies, Santa Clara, CA, USA) (RIN ≥ 7.0). Poly(A)-tailed mRNA was purified using oligo(dT)-attached magnetic beads (Oligotex mRNA Kit, Qiagen, Hilden, Germany). Reverse transcription performed with random hexamer primers and M-MuLV Reverse Transcriptase (Promega, Madison, Wisconsin, WI, USA). cDNA converted to double-stranded DNA using DNA Polymerase I and RNase H. End repair and adapter ligation of cDNA fragments. Size selection via agarose gel purification (target insert size: 300–500 bp). PCR amplification of adapter-ligated cDNA (15 cycles). The transcriptome data generated in this study have been deposited in the NCBI Short Read Archive (SRA) database under the accession number PRJNA1293887.

### 2.5. Illumina Sequencing, Assembly, and Annotation

Paired-end sequencing (150 bp) was performed on the cDNA libraries of tolerant (FS) and susceptible (HA) strains using the Illumina NovaSeq 6000 platform (Illumina, San Diego, CA, USA) with Sequencing by Synthesis (SBS) technology. Adapter sequences and low-quality reads were trimmed using in-house Perl scripts. Clean reads were evaluated for quality based on: Q20/Q30 scores, GC content and duplication levels. High-quality reads were assembled de novo using Trinity (v2.15.1) with default parameters. Contig redundancy was reduced via CD-HIT-EST (identity threshold: 0.95). Contig redundancy was reduced via CD-HIT-EST (identity threshold: 0.95). Assembled transcripts were annotated against multiple databases: NCBI non-redundant protein database (Nr), nucleotide sequence database (Nt), protein family database (Pfam), Clusters of Orthologous Groups (KOG/COG), Swiss-Prot protein database, KEGG Orthology (KO), and Gene Ontology (GO) resources.

### 2.6. Differential Expression Analysis and RT-qPCR Validation

Gene expression levels were quantified using Fragments Per Kilobase of transcript per Million mapped reads (FPKM). Differential expression analysis ((FDR < 0.01) and |log_2_FC| ≥ 2) was performed in R (v4.2.3) [22]. GO enrichment: Conducted using the GOseq R package. KEGG pathway analysis: Performed via KOBAS to identify significantly enriched metabolic/signaling pathways [23,24]. 10 differentially expressed genes (DEGs) were associated with insecticide resistance in the resistant strain.

Total RNA was extracted from newly emerged females (tolerant/susceptible strains) using TRIzol. gDNA removal and reverse transcription were performed using the PrimeScript™ RT Reagent Kit with gDNA Eraser (Takara, Beijing, China). Reaction mix (20 μL total): 1.6 μL cDNA, 10 μL TB Green Premix Ex Taq II (Takara), 0.8 μL each forward/reverse primer (10 μM) and 6.8 μL RNase-free ddH_2_O. Cycling protocol: 95 °C for 30 s (initial denaturation), 39 cycles of: 95 °C for 5 s, 57 °C for 30 s, 72 °C for 30 s. Melt curve analysis (65–95 °C, increment 0.5 °C/5 s). Relative expression levels of DEGs were calculated using the 2^−ΔΔCt^ method. The superoxide dismutase gene (SOD) served as the internal reference [25], as it has been previously validated to exhibit stable expression in *T. dendrolimi* under similar experimental conditions. The primer pairs for both target DEGs and SOD were designed using Primer Premier 5.0 (Table S1). Three biological replicates were analyzed, with each replicate consisting of three technical repetitions.

### 2.7. Data Analysis

Statistical analyses were performed using SPSS 26.0 (IBM, Armonk, NY, USA) and Microsoft Excel 2019 (Microsoft, Redmond, MA, USA). The concentration-mortality data for the HR strain were subjected to probit analysis using SPSS software to calculate the LC_50_ value, its 95% confidence interval (CI), and the regression equation. ANOVA model was employed for analysis and Tukey’s HSD or Dunnett’s multiple comparison method was used for significant difference test of means (*p* < 0.05). RT-qPCR validation tests were tested Chi-square test. Graphical representations were generated with GraphPad Prism 8.0.2 (GraphPad Software, San Diego, CA, USA).

## 3. Results

### 3.1. Toxicity Bioassay

Based on the probit analysis of the HR strain (Table 2), a discriminating dose of 0.1 mg/L imidacloprid was selected for subsequent sensitivity assays across 17 geographic strains of *T. dendrolimi*.

**Figure 1:**
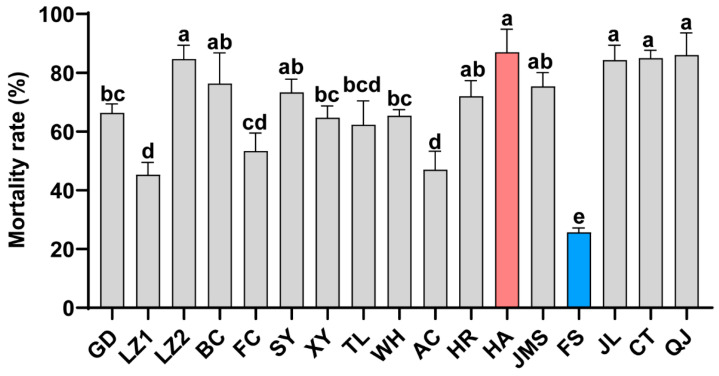
Mortality at 24 h was recorded for 17 geographic strains of *T. dendrolimi* following 1 h of exposure to a screening dose of 0.1 mg/L imidacloprid. The Y axis represents the mortality rate. Error bars represent the standard deviation (SD) of the three replicates. Lower case letters indicate significant differences among treatments (one-way *ANOVA* and Tukey HSD comparison, *F*_16,34_ = 27.60, *p* < 0.0001).

The imidacloprid resistance of 17 geographic strains of *T. dendrolimi* was assessed using a film-residue bioassay. The results revealed significant variations in susceptibility among the strains (Figure 1). After 1 h of imidacloprid exposure, the 24 h mortality rate of the HA strain (87%) was significantly higher than that of the FS strain (25.7%). The mortality rates of the remaining strains, in descending order, were as follows: QJ (86%) > CT (85%) > LZ2 (84.7%) > JL (84.3%) > BC (76.3%) > JMS (75.3%) > SY (73.3%) > HR (72%) > GD (66.3%) > WH (65.3%) > XY (64.7%) > TL (62.3%) > FC (55.3%) > AC (47%) > LZ1 (45.3%). Based on these findings, the FS strain was identified as the tolerant strain, whereas the HA strain was classified as the susceptible strain.

### 3.2. Biological Assays

**Figure 2:**
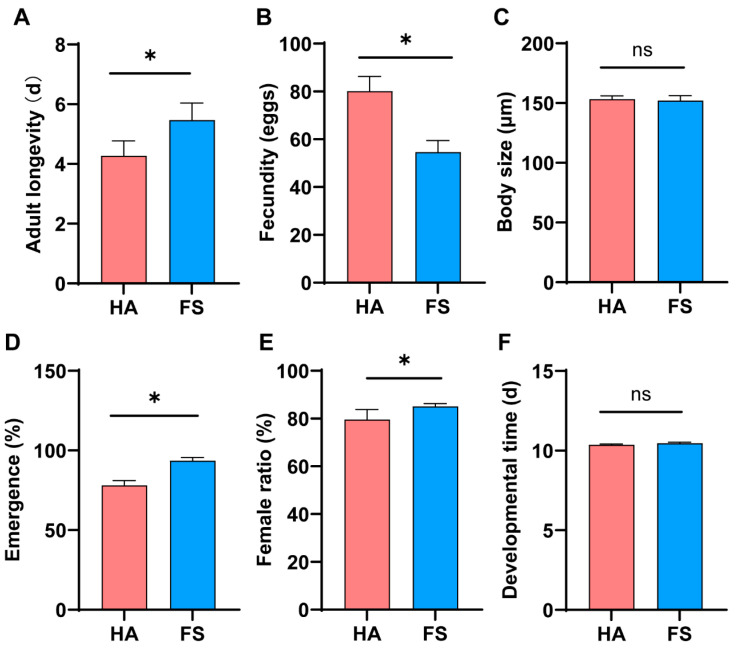
Biological effects of imidacloprid stress on the most tolerant (FS) and most susceptible (HA) of *T. dendrolimi* following exposure to a screening dose of 0.1 mg/L imidacloprid. Error bars represent the standard deviation (SD). Paired *t*-test, adult longevity: *t* = 6.131, df = 28, *p* < 0.01; fecundity: *t* = 14.40, df = 38, *p* < 0.01; body size: *t* = 0.9409, df = 30, *p* < 0.01; emergence: *t* = 19.52, df = 38, *p* < 0.01; female ratio: *t* = 5.631, df = 38, *p* < 0.01; developmental time: *t* = 5.810, df = 38, *p* > 0.05. *n* = 30. The asterisk (*) indicates *p* < 0.05, The ns indicates *p* > 0.05.

The biological parameters of the tolerant strain (FS) and the susceptible strain (HA) were evaluated following treatment using the film-residue method. The results showed that FS exhibited significantly longer adult longevity (5.467 ± 0.568 d), higher emergence rate (93.60 ± 1.90%), and greater female ratio (85.10 ± 1.20%) compared to HA (4.267 ± 0.502 d; 78.10 ± 3.00%; 79.60 ± 4.20%). In contrast, HA (80.100 ± 6.231 eggs) demonstrated significantly higher fecundity than FS (54.600 ± 4.890 eggs). No significant differences were observed between the two strains in terms of body size (FS: 152.144 ± 4.181 μm; HA: 153.331 ± 2.826 μm) or developmental time (FS: 10.467 ± 0.060 d; HA: 10.363 ± 0.053 d) (Figure 2).

### 3.3. Transcriptome Overview

Sequencing results demonstrated high data reliability, with filtered reads averaging 36,855,896 for FS and 32,037,287 for HA. The mean Q20 scores were 98.47% (FS) and 97.91% (HA), while Q30 values averaged 94.71 and 93.08, respectively. Both strains exhibited GC contents exceeding 40%, collectively indicating high sequencing quality (Table S2).

**Figure 3:**
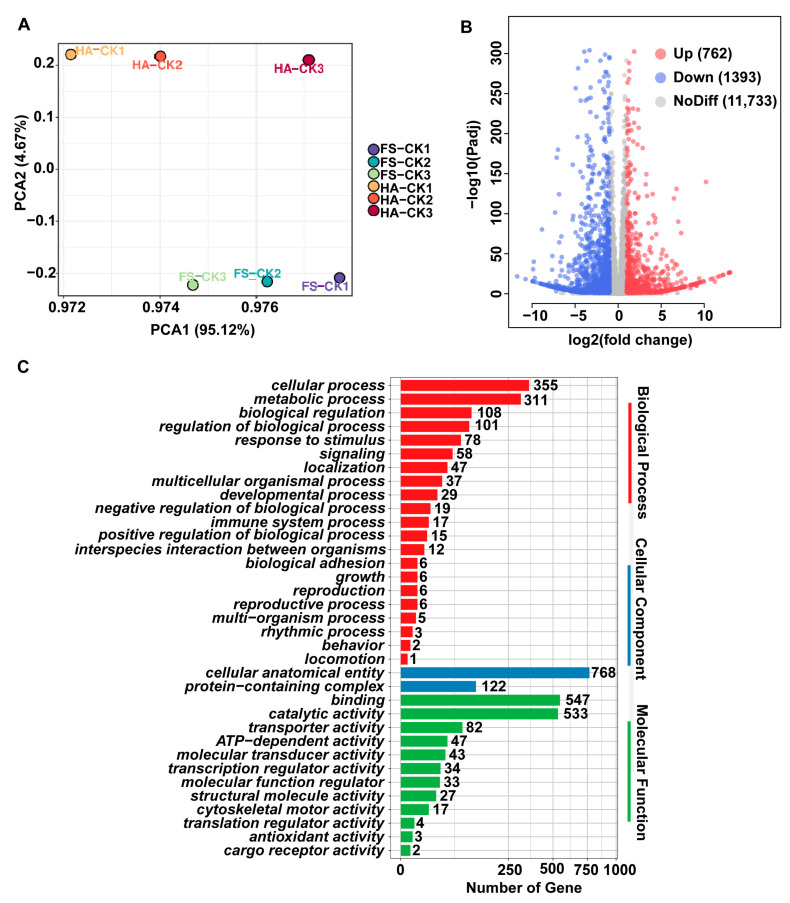
Transcriptome sequencing of the tolerant (FS) and susceptible (HA) strains of *T. dendrolimi*. (**A**) Principal component analysis (PCA). (**B**) The volcano plot of differentially expressed genes (*FS*-CK vs. *HA*-CK) in the transcriptome. The X axis and Y axis represent Log2 fold-change (Log2FC) differences between the contrasted groups and statistical significance as the negative Log10 of adjusted *p*-value (s-value), respectively. (**C**) Gene Ontology (GO) enrichment analysis.

Principal component analysis (PCA) revealed significant clustering patterns between FS and HA (*p* < 0.05) (Figure 3A). A total of 2115 DEGs were identified between the strains, comprising 762 up-regulated and 1393 down-regulated genes (Figure 3B). Gene Ontology (GO) enrichment analysis demonstrated distinct functional categorization of these DEGs: Biological processes: 355 genes were enriched in cellular processes, followed by 311 genes in metabolic processes; Cellular components: the “cell anatomical entity” term showed the most significant enrichment (768 genes); Molecular functions: binding-related (547 genes) and catalytic activity-related (533 genes) terms predominated (Figure 3C).

**Figure 4:**
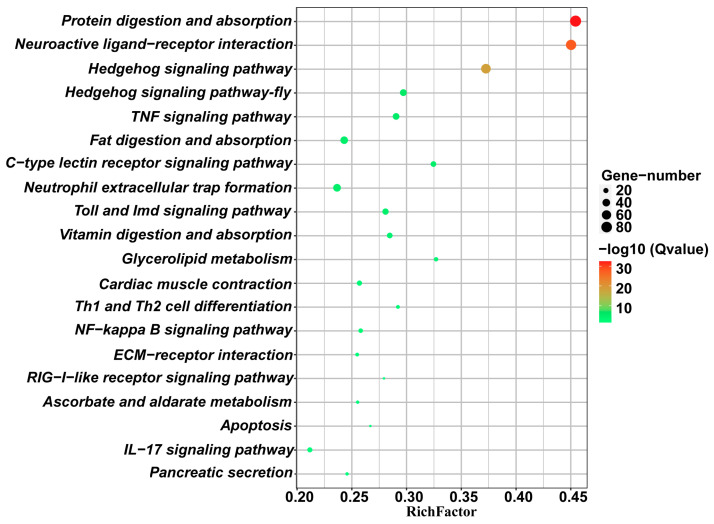
KEGG pathway enrich of FS vs. HA differentially expressed genes.

KEGG enrichment analysis of DEGs in *T. dendrolimi* revealed significant enrichment in multiple metabolic and signaling pathways. The most enriched pathways were “Protein digestion and absorption” and “Neuroactive ligand-receptor interaction”, indicating their potential roles in imidacloprid response. Immune-related pathways, including “TNF signaling pathway,” “C-type lectin receptor signaling pathway,” “IL-17 signaling pathway,” and “NF-kappa B signaling pathway,” were also significantly enriched, suggesting involvement of immune regulation in insecticide defense. The high RichFactor values and statistical significance (−log10(Qvalue)) demonstrate the biological relevance of these pathways in imidacloprid resistance. Metabolic pathways such as “Fat digestion and absorption,” “Vitamin digestion and absorption,” and “Glycerolipid metabolism” showed notable enrichment, implying metabolic adaptations in the tolerant strain (FS). Additionally, “Hedgehog signaling pathway” and “Apoptosis” were enriched, reflecting potential alterations in cellular development and programmed cell death. These results indicate a multifaceted transcriptional response in *T. dendrolimi*, involving digestion, immunity, metabolism, and signal transduction under insecticide stress (Figure 4).

### 3.4. RT-qPCR Validation

**Figure 5:**
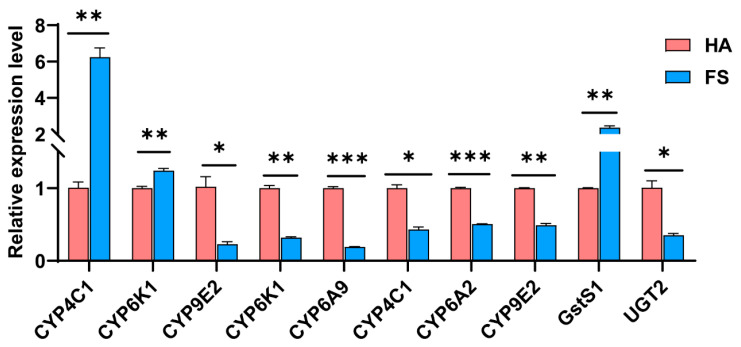
RT-qPCR validation of candidate DEGs in the imidacloprid-tolerant (FS) versus the susceptible (HA) strain of *T. dendrolimi*. Paired *t*-test, *CYP4C1*: *t* = 8.923, df = 2, *p* < 0.01; *CYP6K1*: *t* = 16.92, df = 2, *p* < 0.01; *CYP9E2*: *t* = 4.593, df = 2, *p* < 0.05; *CYP6K1*: *t* = 22.35, df = 2, *p* < 0.001; *CYP6A9*: *t* = 39.20, df = 2, *p* < 0.001; *CYP4C1*: *t* = 7.069, df = 2, *p* < 0.05; *CYP6A2*: *t* = 36.42, df = 2, *p* < 0.001; *CYP9E2*: *t* = 17.02, df = 2, *p* < 0.01; *GstS1*: *t* = 13.24, df = 2, *p* < 0.01; *UGT2*: *t* = 5.567, df = 2, *p* < 0.05. *n* = 3 biological replicates. The asterisk (*) indicates *p* < 0.05, The two asterisk (**) indicates *p* < 0.01, The three asterisk (***) indicates *p* < 0.001.

Our findings suggest that altered expression levels of key detoxification and oxidase genes in *T. dendrolimi* may constitute a crucial mechanism underlying imidacloprid resistance. From the transcriptome sequencing data, we identified 10 DEGs related to detoxification enzymes or oxidases for subsequent validation (Table S3). Compared with the susceptible HA strain, the tolerant FS strain exhibited significant upregulation of *CYP4C1*, *CYP6K1*, and *GstS1*, with *CYP4C1* showing the highest expression level. These results were consistent with the transcriptome sequencing trends, strongly suggesting that these three genes participate in the development of imidacloprid resistance in *T. dendrolimi* (Figure 5).

## 4. Discussion

### 4.1. Toxicity Bioassay and Resistance Variability Among Geographic Strains

The toxicity bioassay established an LC_50_ value of 0.075 mg/L for imidacloprid in the HR strain of *T. dendrolimi*. This value served as the basis for selecting a standardized discriminating dose of 0.1 mg/L, which was subsequently used to assess the susceptibility of 17 geographic strains. The results demonstrated pronounced variability in mortality rates, ranging from 25.7% in the FS strain to 87% in the HA strain. The observed resistance patterns likely reflect genetic adaptation, as all strains were maintained for over 20 generations under identical, insecticide-free laboratory conditions prior to testing, which would have eliminated any non-genetic, acclimation effects from the field. Previous studies have documented significant interspecific variation in pesticide sensitivity among *Trichogramma* wasps. Yang et al. [13] reported that *T. evanescens* exhibited the lowest susceptibility to pyrethroids, whereas *T. dendrolimi* showed the most pronounced response. Likewise, Xu et al. [11] showed *T. ostriniae* was more sensitive to imidacloprid than *Encarsia formosa*. According to Wang et al. [12], a film-residue bioassay revealed differential toxicity of neonicotinoids and macrocyclic lactones to four *Trichogramma* wasps.

Liu [26] showed that neonicotinoid insecticides were toxic to three *Trichogramma* wasps: *T. dendrolimi*, *T. ostriniae*, and *T. confusum*. The study found that nitenpyram was most acute toxic to both *T. dendrolimi* and *T. Ostriniae*, dinotefuran was the most toxic neonicotinoid to *T. confusum*. In this study, the FS strain was classified as tolerant because it had the lowest mortality rate and the HA strain was deemed susceptible because it had the highest mortality rate.

The resistance patterns that were seen could reflect local selection due to more frequent use of imidacloprid [27]. The differences in resistance between areas mean the areas need their own monitoring and managements. In addition, identifying the resistant and susceptible strains creates a useful model for studying the genetic and physiological basis of imidacloprid resistance in *T. dendrolimi*.

### 4.2. Biological Trade-Offs Associated with Resistance

Parasitic wasps’ eco-friendly characteristics are determined by the ecological and physiological compatibility of interactions between parasitoids and host [28]. Our study revealed a clear fitness trade-off associated with imidacloprid tolerance in *T. dendrolimi*. When compared to the most susceptible strain (HA), the most tolerant strain (FS) exhibited significantly extended adult longevity and higher emergence rate, but at the cost of a substantial reduction in fecundity (Figure 2). No significant differences were observed in body size or developmental time. The results match the concept of fitness trade-off in which resistance mechanisms provide a survival advantage in the face of insecticide exposure but incur a cost in other life-history traits [17,29]. The fact that the FS strain shows lower fecundity suggests a potential re-allocation of energy towards detoxification and survival processes, which are metabolically demanding. Therefore, trade-offs play a key role in predicting the dynamics of resistance evolution in field populations [30,31].

### 4.3. Transcriptomic Insights into Resistance Mechanisms

Transcriptome sequencing revealed high-quality data. PCA was able to distinguish both strains which further supported the reliability of transcriptional differences. The GO enrichment analysis highlighted functional categories that may be involved in resistance. The fact that digestion and metabolism pathways are enriched suggests that *T. dendrolimi* can involve the processing of nutrients and allocation of energy for detoxification. The major involvement of immune and signalling pathways suggests a concerted defence mechanism against chemical stress that may lead to survival adaptation [32,33]. Significantly, the detoxification and oxidase-related genes are often related to insecticide resistance [34,35,36]. Validation through RT-qPCR confirmed the transcriptome data, showing up-regulation of the genes *CYP4C1*, *CYP6K1*, and *GstS1* in the FS strain. The characteristic expression of three FS strain genes suggests their key functions in imidacloprid detoxification. The greatly increased activity of three genes has been recorded in previous reports of neonicotinoid resistance in other insects. For example, Cytochrome P450 monooxygenases (*CYP4C1* and *CYP6K1*) are known to metabolize neonicotinoids [37,38,39], while the glutathione S-transferases (*GstS1*) conjugates reactive intermediates for excretion [40,41].

### 4.4. Implications for Resistance Management and Biological Control

The identification of tolerant *T. dendrolimi* strains and the underlying mechanisms opens a dialogue on their potential application in IPM. While deploying resistant parasitoids could theoretically improve pest control efficacy in crops where neonicotinoid residues are present [42], this approach warrants cautious consideration. A primary concern, is that reliance on resistant natural enemies should not inadvertently justify or promote the continued extensive use of broad-spectrum insecticides, which are detrimental to a wider range of non-target organisms and ecosystem services [28,43]. Instead, the value of this research may lie more in conservation biological control. Understanding resistance mechanisms allows for the development of diagnostic tools to monitor susceptibility in field populations [44,45]. This knowledge can guide the augmentation of native resistant populations rather than solely relying on the release of lab-selected strains. Future research should focus on evaluating the ecological fitness and stability of resistance in complex field environments, and on designing IPM protocols that prioritize the preservation of overall biodiversity and ecosystem health.

In short, this study helps understand the phenotypic and molecular basis of imidacloprid resistance in *T. dendrolimi*. The tolerant FS strain has distinct biological trade-offs including less fecundity but greater survival when exposed to insecticide. According to transcriptomic and RT-qPCR analyses, *CYP4C1*, *CYP6K1*, and *GstS1* were found to play a role in detoxification and can be useful targets for resistance monitoring and management. The findings will help in enhancing the understanding of resistance evolution and strategies for sustainable pest control.

## Figures and Tables

**Table 1:** The collection sites of *Trichogramma dendrolimi*.

NO.	Collection Sites	Collection Times	Host	Strain Name	Latitude and Longitude
1	Shen yang (Liao ning)	2016.7	*Chilo suppressalis*	SY	41.7143° N, 123.4510° E
2	Liao zhong 1 (Liao ning)	2018.8	*Dictyoploca japonica*	LZ1	41.5126° N, 122.7282° E
3	Liao zhong 2 (Liao ning)	2018.8	*Dictyoploca japonica*	LZ2	41.5126° N, 122.7282° E
4	You Yan (Liao ning)	2016.7	*Dictyoploca japonica*	XY	40.2877° N, 123.2719° E
5	Feng cheng (Liao ning)	2020.8	*Fentonia ocypete*	FC	40.4500° N, 124.0637° E
6	Huan ren (Liao ning)	2016.7	*Dictyoploca japonica*	HR	41.2681° N, 125.3487° E
7	Fu shun (Liao ning)	2019.8	*Dendrolimus*	FS	41.8692° N, 123.9241° E
8	Chang tu (Liao ning)	2019.8	*Chilo suppressalis*	CT	42.7849° N, 124.1054° E
9	Ji lin	2019.8	*Chilo suppressalis*	JL	43.8843° N, 125.3180° E
10	Bai cheng (Ji lin)	2019.8	*Chilo suppressalis*	BC	45.6156° N, 122.8360° E
11	Wu han (Hu bei)	2018.8	*Antherea pernyi egg collection*	WH	30.6041° N, 114.2653° E
12	Hong an (Hu bei)	2018.8	*Antherea pernyi egg collection*	HA	31.2904° N, 114.6131° E
13	Guang dong	2018.8	*Antherea pernyi egg collection*	GD	23.1301° N, 113.2592° E
14	Tong liao (Nei meng gu)	2019.8	*Chilo suppressalis*	TL	42.7334° N, 121.7937° E
15	A cheng (Hei long jiang)	2019.8	*Chilo suppressalis*	AC	45.5362° N, 126.9694° E
16	Jia mu si (Hei Long jiang)	2019.8	*Dendrolimus*	JMS	46.8087° N, 130.3734° E
17	Qian jin farm (Hei Long jiang)	2019.8	*Dendrolimus*	QJ	47.3953° N, 126.808° E

**Table 2:** Toxicity assessment of imidacloprid on *Trichogramma dendrolimi* (HR).

Strain	N	Regression Equation	Slope ± SE	R^2^	LC_50_ (mg/L)	95% CI
HR	20	Y = 1.17x + 6.32	1.17 ± 0.22	0.969	0.075	0.020–0.274

Note: N, number of individuals tested; SE, standard error; CI, confidence interval.

## Data Availability

The original contributions presented in this study are included in the article/Supplementary Material. Further inquiries can be directed to the corresponding author.

## Supplementary Materials

The following supporting information can be downloaded at: https://www.mdpi.com/article/10.3390/insects16101038/s1, Table S1. Primers used for the RT-qPCR; Table S2. Sequencing data quality assessment.; Table S3. FS-CK VS HA-CK detoxification metabolism related genes.
