# Supplementary material for: Fitness Trade-Offs and Potential Metabolic Resistance Mechanisms in Geographically Distinct Strains of Trichogramma dendrolimi: Implications for Imidacloprid Resistance Management
Source: Insects. 2025 Oct 9;16(10):1038. doi: 10.3390/insects16101038 (PMC12565574; doi:10.3390/insects16101038)
Supplement: Supplementary file 1 [file insects-16-01038-s001.zip › insects-3876120-supplementary.pdf]

Supplementary materials for

Fitness trade-offs and metabolic resistance mechanisms in *Trichogramma dendrolimi*: implications for imidacloprid resistance management

List of contents:

Table S1. Primers used for the RT-qPCR.

Table S2 Sequencing data quality assessment.

Table S3 FS-CK VS HA-CK detoxification metabolism related genes.

TableS1 Primers used for the RT-qPCR

Gene Names	Prime names	Sequences
Tde000636	0636F1	GGCCTTGGCCCTTGTAATT
	0636R1	ACGTCATCCGGGTGTTGTA
Tde004448	4448F1	TACTGGCGACGAAGAGGAG
	4448R1	TTGCTTGATAATTTCTGGACTGC
Tde008577	8577F1	TTCGATCCCGATCGTTATT
	8577R1	TCATGGGTGAACTGGTCTTT
Tde010868	10868F1	CAATGTCCCTTGACGAAAC
	10868R1	ACATATTCTTGGCCCATCT
Tde008668	8668F1	AAGATTATACGATACCAGGCACG
	8668R1	CGCCGAACGGTAAGAAATA
Tde008535	8535F1	AGAAACGGACGATTATTAG
	8535R1	TTACGACGTGTAAATTCAA
Tde009543	9543F1	GTCAGGCTCGTTCTTGTCA
	9543R1	AGTTCGTCTCCGATTTCTTT
Tde005938	5938F1	CTCACTTCACCGATCACAAG
	5938R1	TCGAACATTGCCCTCATTT
Tde009151	9151F1	TTTTCGATCTGCCGATACTTG
	9151R1	GCGAGCGACTTGATTCTCA
Tde009184	9184F1	CTCGCTCATGGCCCTGGAGT
	9184R1	GCGTGATGATAGATCGCCTCC
GAPDH	GAPDHF1	TACGACGAGATCAAGGCCAA
	GAPDHR1	ATGACACGGGATGAGTAGCC

TableS2 Sequencing data quality assessment

Sample	Clean Reads	Clean Base	Q20(%)	Q30(%)	GC (%)
FS-CK1	39,158,446	11,747,533,800	98.43	94.60	40.44
FS-CK2	37,304,713	11,191,413,900	98.44	94.60	40.56
FS-CK3	34,104,528	10,231,358,400	98.54	94.93	40.53
Average	36,855,896	11,056,768,700	98.47	94.71	40.51
HA-CK1	33,361,423	10,008,426,900	98.51	94.87	40.35
HA-CK2	28,884,793	8,665,437,900	98.52	94.89	40.76
HA-CK3	33,865,646	10,159,693,800	96.71	89.48	40.52
Average	32,037,287	96,111,862,000	97.91	93.08	40.54

Table S3 FS-CK VS HA-CK detoxification metabolism related genes

Gene_name	Gene ID	Log_2FC	NR_annotation
CYP4C1	Tde000636	8.958	cytochrome P450 4C1-like
CYP6K1	Tde004448	2.595	cytochrome P450 6k1-like
CYP9E2	Tde008577	-11.733	cytochrome P450 9e2-like
CYP6K1	Tde010868	-4.430	cytochrome P450 6k1-like
CYP6A9	Tde008668	-3.142	cytochrome P450 6a9-like
CYP4C1	Tde008535	-2.806	cytochrome P450 4C1-like
CYP6A2	Tde009543	-2.774	cytochrome P450 6a2-like
CYP9E2	Tde005938	-2.508	cytochrome P450 9e2-like
GstS1	Tde009151	2.198	Glutathione S-transferase
UGT2	Tde009184	-4.226	UDP-glucosyltransferase 2
